# Intratumorally delivering cytokines via a universal and modular strategy

**DOI:** 10.1186/s43556-023-00117-3

**Published:** 2023-02-15

**Authors:** Xinping Zhang, Fu-Gen Wu

**Affiliations:** State Key Laboratory of Bioelectronics, School of Biological Science and Medical Engineering, Southeast University, Nanjing, 210096 China

A universal and modular strategy for intratumoral delivery of cytokines was recently reported by Agarwal et al. in an article published in *Nature Biomedical Engineering* [1].

Cytokines are messenger molecules that mediate intercellular communication, and play an important role in host antitumor immunity. Various categories of cytokines are implicated in tumor development, progression, and metastasis, mainly including interleukins (ILs), interferons (IFNs), chemotactic cytokines (chemokines), growth factors, and some members of the tumor necrosis factor (TNF) superfamily. Some cytokines can directly inhibit tumor growth, such as transforming growth factor-β (TGF-β) and IFN-α, while others (e.g., IFN-γ, IL-2, IL-12, and IL-15) achieve the antitumor effect by enhancing the cytotoxic activity of lymphocytes [2]. Specially, IFN-γ can also improve the cytotoxic activity of myeloid cells. Based on these antitumor effects, the applications of cytokines in cancer treatments have been extensively investigated during the past few decades. However, it has been reported that cytokines usually have only limited antitumor therapeutic efficacy and can cause severe adverse reactions when administered systemically (intravenously or subcutaneously) [3]. Therefore, developing efficient cytokine delivery strategies has become a research focus. Since the main targets of cytokines are tumor-associated immune cells, the intratumoral (*i*.*t*.) administration of cytokines may be expected to improve the therapeutic effect and mitigate systemic toxicity. In addition, with the development of interventional techniques, researchers no longer have to concern about the feasibility of *i*.*t*. dosing in most locations of lesions, suggesting the broad prospect of *i*.*t*. cytokine-based therapy.

Notwithstanding, the rapid escape of soluble cytokines from the tumor after *i*.*t*. administration of the cytokines invariably leads to unsatisfactory therapeutic effect and systemic toxicity. Recently, Wittrup, Irvine, and coworkers developed a universal and modular strategy for the *i*.*t*. delivery of high-dose cytokines, realizing the long retention of cytokines within tumor sites for weeks after injection [1] (Fig. 1). The authors took advantage of the ligand exchange between phosphorylated proteins and the surface hydroxyls of aluminum hydroxide (alum) to form alum-anchored cytokines by simply mixing alum and cytokines modified by a phosphorylated alum-binding peptide (ABP) tag. As a vaccine adjuvant approved by the US Food and Drug Administration (FDA), alum has been safely used in human for a long time. Besides, the microscale aggregates of nanoscale rod-shaped nanocrystals in alum can build a physical depot at the administration site, which is able to persist for several weeks, thus achieving the long retention and sustained release of cytokines.

**Fig. 1:**
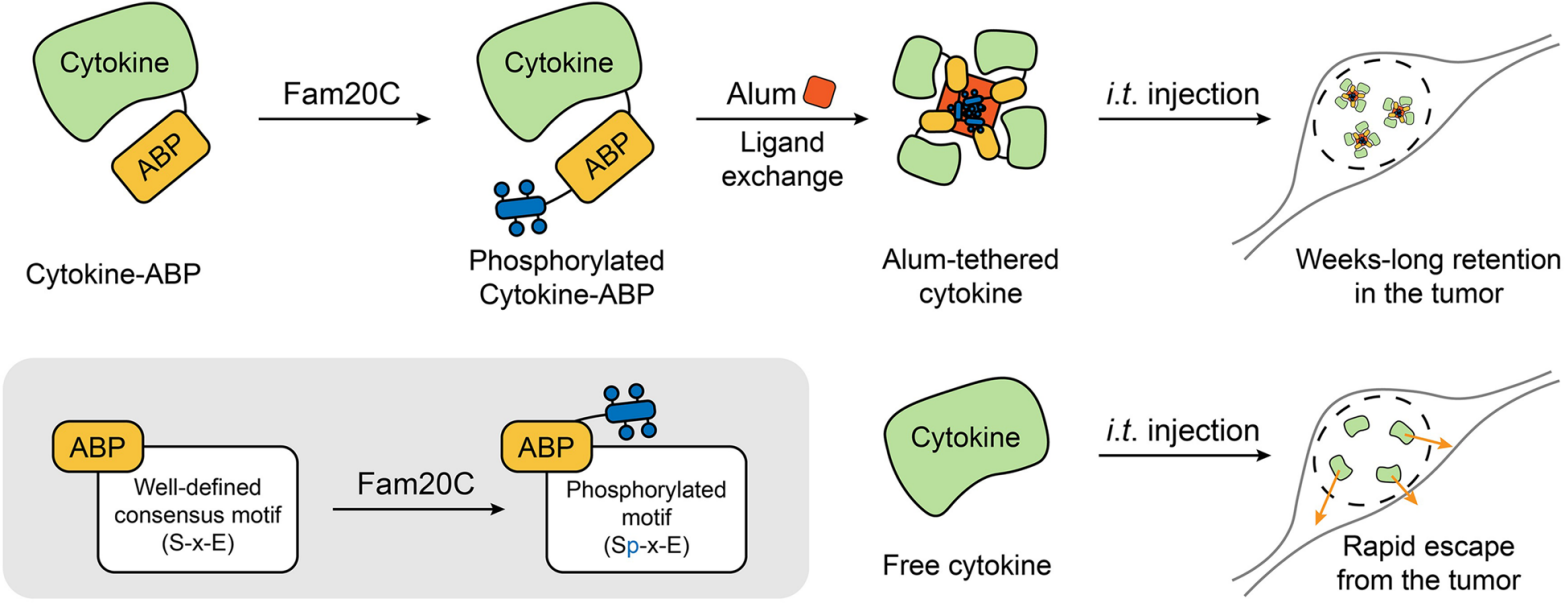
Fabrication of alum-tethered cytokines and their capacity to realize prolonged tumor retention after *i*.*t*. injection

To produce the phosphorylated ABP-labeled cytokines, Fam20C, a kind of kinase in charge of the phosphorylation of most of the mammalian cell-secreted phosphoproteome, was engineered with a KDEL C-terminal sequence, which is an endoplasmic reticulum retention signal. Additionally, an initial set of ABPs with S-x-E motifs in which serines can be recognized and phosphorylated by Fam20C, was designed and fused to diverse therapeutic cytokines, including a single-chain form of IL-12, mouse serum albumin-fused IL-2, and an IL-15 superagonist complex with the IL-15α chain (IL-15sa). Thus, when coexpressed together with Fam20C-KDEL in cell expression platforms, cytokines modified with ABPs can be specifically phosphorylated by Fam20C during their secretion from mammalian cells. The resulting proteins were then subjected to immobilized metal affinity chromatography and subsequent anion exchange chromatography treatments for purification. The obtained ABP-fused cytokines exhibit stable alum binding potency while still maintaining activity. Specifically, alum-anchored IL-12 exhibited a remarkably increased *i*.*t*. retention compared with free IL-12, with no observed systemic toxicity. Furthermore, a single dose of alum-tethered IL-12 induced a robust IFN-γ-mediated synergism between adaptive and innate immunocytes, as well as notable activation of antigen-presenting cells in draining lymph nodes, thereby producing strong systemic antitumor effects in poorly immunogenic tumor models when used in conjunction with checkpoint blockade therapy. The inhibition of distant untreated tumor growth and lung metastasis was also achieved.

Actually, Wittrup’s group has previously reported an approach of anchoring cytokines to lumican (a collagen-binding protein) for enhancing local retention after *i*.*t*. injection [4]. Although this strategy can also reduce systemic toxicities of free cytokines while enhancing therapeutic effects, the therapeutic performance of this strategy is dose-limited by the collagen amount within the tumor, which is variable in different patients and tumors. By contrast, the *i*.*t*. administration of alum-bound cytokines is a tumor-agnostic approach for realizing the concurrent enhancement of local and systemic antitumor responses. Furthermore, the site-specific binding of cytokines to the solid surface of alum may also enhance the stability and protect the cytokines from degradation by endogenous proteases. Additionally, by optimizing the sequence of the linker between target cytokine and ABP, the location of ABP in the protein, and the design of ABP, this delivery strategy can be widely applied to diverse cytokines, and has been successfully utilized to *i*.*t*. injection of IFNs as well [5].

Although the enclosure of cytokines within material depots (e.g., hydrogels, polymeric microspheres, and mesoporous silica nanoparticles) can prevent their rapid leakage, it is still challenging to tune the release rate of cytokines to coordinate with their *i*.*t*. consumption rate. Intratumorally administering cytokines in the alum-anchored format can obviate this issue. However, to fuse with ABPs, the single-chain forms of cytokines are generally used, which may lead to different degrees of activity loss of cytokines. Besides, the binding of cytokines to alum has an influence on the activity of some cytokines; for instance, phosphorylated IL-2–ABP and IL-12–ABP showed a 4-fold loss in activity when tethered to alum [1]. On the other hand, it is also worth noting that the acidic and hypoxic tumor microenvironment (TME) may disrupt cytokine signaling, resulting in a markedly compromised antitumor immunity of cytokine-based therapy. Therefore, it will be attractive to explore TME-resistant cytokine variants for the application of this modular delivery strategy. In addition, the probable side effects including chronic inflammations and autoimmune reactions caused by long-term body retention of residual cytokines after cancer treatment need to be carefully investigated. The long-term metabolic process of alum–cytokine requires to be studied. The addition of a TME-responsive module (e.g., a pH-responsive linker or a hypoxia-responsive linker) is expected to provide a promising tool for manipulating spatiotemporal cytokine release/exposure profile, which will further expand the therapeutic window. Furthermore, it is crucial to find the optimal combination of the *i*.*t*. administration of alum-anchored cytokines and a systemic therapeutic modality such as immune checkpoint blockade therapy or chimeric antigen receptor T (CAR T) cell therapy for achieving an improved therapeutic outcome. Overall, this modular method for safe *i*.*t*. delivery of cytokines introduced by Wittrup, Irvine, and coworkers may offer inspirations for the potential clinical applications of cytokines.

## Data Availability

Not applicable.

## Abbreviations

ABP: Alum-binding peptide
CAR T: Chimeric antigen receptor T
FDA: US Food and Drug Administration
IFN-α: Interferon-α
IFN-γ: Interferon-γ
IL-2: Interleukin-2
IL-12: Interleukin-12
IL-15: Interleukin-15
TGF-β: Transforming growth factor-β
TME: Tumor microenvironment
